# Educational outcomes of the first longitudinal integrated clerkship program in Israel

**DOI:** 10.5116/ijme.60f5.ef72

**Published:** 2021-08-26

**Authors:** Ron Eshel, Reli Hershkovitz, Amos Katz, Jacob Orkin, Shimon Amar

**Affiliations:** 1 Division of Anesthesia, Intensive Care, and Pain Management, Tel Aviv Medical Center, Sackler Faculty of Medicine, Tel-Aviv University, Tel-Aviv, Israel; 2 Faculty of Health Sciences, Goldman Medical School, Ben-Gurion University of the Negev, Beer-Sheva, Israel

**Keywords:** Educational outcomes, first longitudinal integrated clerkship, Israel

## To the Editor

The longitudinal integrated clerkship (LIC), an approach to clinical medical education that is gaining in popularity, represents a transformative trend that emphasizes relationships between medical students, patients, and physicians as a means of shaping the educational experience.^1^^-^^3^ This variation on the standard sequential, time-limited specialty-specific 'block' rotations, often referred to as traditional block rotations (TBRs), aims to enhance students' experience of continuity of care, doctor-patient relationships, and academic supervision.^4^^-^^6^

The Joyce and Irving Goldman Medical School of Ben-Gurion University of the Negev is the first Israeli institution to adopt the LIC model in their current clinical curricula. In our study, we aimed to quantify and describe the effect of a clinical clerkship undertaken according to the LIC model in comparison to TBRs, by sixth-year medical students in the Faculty of Health Sciences at Ben-Gurion University of the Negev.

The program was presented to the sixth-year class of medical students as an alternative to TBR and all students were encouraged to join. Participation in the LIC program was entirely voluntary, without reward, and had no effect on a student's final grade. Following a personal interview and two preliminary meetings with each of the 12 students that applied, a final group of eight students was selected by the program's directors based on their commitment and enthusiasm.

Regardless of their clinical group assignment, during the study period, all students completed their core rotations in internal medicine and pediatrics, with a combination of training in wards, inpatient clinics, emergency departments, and ambulatory care. Clinical rounds were performed under the faculty's partnership with Soroka University Medical Center, a leading tertiary medical and university center.

The LIC program operated under an established prearranged weekly schedule that included engagement in the clinical routine of selected internal and pediatric wards, specific subject-oriented academic sessions, and active participation in the community clinic of a selected general practitioner who was directly responsible for guiding and supervising the students. TBR students, on the other hand, participated in their assigned ward's daily routine of grand rounds, patient examination, etc., with no additional academic sessions performed.

Data collection was performed via anonymous and identical electronic questionnaires distributed to the entire class, LIC and TBR students alike, before and after clinical rounds. Participation in the questionnaires was entirely voluntary without any form of reward.

During the study period, a total of 36 questionnaires were collected. From the LIC group, seven sets of pre-and post-round forms were collected, with one student failing to perform the pre-rounds survey. Complete pre-and post-round questionnaire sets were collected from seven students in the TBR group, with an additional seven pre-rounds and 14 post-rounds surveys.

Results of surveys collected in the pre-rounds stage demonstrated no statistically significant differences between the groups regarding demographic characteristics, other than a male majority within the LIC group. Overall, both groups expressed similar expectations from their upcoming clerkships. However, the TBR group expressed concern about the ability to ask basic medical questions during their rounds.

At the end of the clinical clerkships, students in the LIC group reported greater fulfillment of their expectation of improving their ability to create meaningful relationships with patients and their families. LIC students expressed a generally positive attitude regarding the LIC clinical clerkship model. On the other hand, TBR students were more likely to describe their rounds as "boring" or "frustrating".

A comparison of the groups' academic performance on their national medical license exams at the end of the school year found that students participating in the LIC model were, on average, more proficient than their classmates. These findings highlight the academic non-inferiority of the LIC program and are aligned with the extensive review performed by Walters et al. 4 that showed that LIC students achieve academic results equivalent to, and in some cases better than, their TBR peers.

Results from the questionnaires completed at the end of the clinical rounds demonstrate a significant difference in satisfaction between the groups. Students from the LIC group were less likely to use negative adjectives to describe their clinical clerkships compared to their TBR counterparts. These outcomes are aligned with the general principles at the core of the LIC model, emphasizing an empowering student experience that accentuates the patient-physician relationship. The generally positive assessment was consistent with the participants' final evaluation of the LIC model and its fulfillment of pre-rounds expectations.

An important aspect of the clerkship model emerges when comparing the groups' expectations of learning how patients deal with their disease within the medical system. Despite the lack of a significant difference in the initial survey, at the end of the clerkships, the students that participated in the LIC program described gaining a better understanding of the difficulties endured by patients than did their TBR peers. This result highlights the importance of the LIC model in teaching future physicians about patients' trials and tribulations within the healthcare system.

The findings observed in this study are consistent with previous studies comparing the LIC and TBR models.^7^^-^^10^ Students from the LIC group reported feeling more confident in their ability to connect with patients and perform a thorough examination. We believe this attitude is the result of the close relationships formed between the students and their patients.

Despite the proven international efficacy of the program and the benefits mentioned above, we encountered significant difficulties in integrating the LIC model in the academic curriculum. The involvement of community health facilities requires substantial resources for the recruitment of physicians and students, providing and funding transportation solutions for students, and creating an academic and administrative support system for the entire program. We believe that the success of our pilot can be greatly attributed to the longstanding relationships and cooperation between the institutions involved and their shared commitment to improving medical education and healthcare.

The program's duration can be seen as a limitation of this study. Although it represented a significant portion of the academic year, the nine-week implementation of our LIC model was of limited duration compared to other programs that have been studied around the world.^3^^,^^5^^,^^11^ We believe that this obstacle, along with the relatively small total questionnaires collected, provides a possible explanation for the lack of statistical significance for many of our variables.

At their core, LIC programs aim to improve medical students' education, professional development, and satisfaction. Compared to TBR students, students in the LIC program showed increased clinical confidence and fulfillment. By increasing students' commitment and understanding of both the patients and the medical system, LIC programs worldwide develop physicians who are better prepared and more patient-oriented. We believe that our results reinforce the growing literature supporting LIC programs as credible and effective pedagogical alternatives to traditional models of medical education.

Finally, we wish to share the words of Y.G., one of the students who volunteered to participate in the LIC pilot: "It was an excellent experience. I got to know people in a human way, not from "above" as an official doctor would. The most important thing for me is that the LIC program succeeded in restoring my joy in studying medicine. It reminded me why I came to medical school in the first place."

### Conflicts of Interest

The authors declare that they have no conflict of interest.
